# Surgical approach for the treatment of aortoesophageal fistula combined with dual aortic aneurysms: a case report

**DOI:** 10.1186/1749-8090-8-206

**Published:** 2013-11-04

**Authors:** Rihao Xu, Tiance Wang, Dan Li, Zhicheng Zhu, Shudong Zhang, Chengluan Xuan, Wen Yan, Kexiang Liu

**Affiliations:** 1 Department of Cardiovascular Surgery, Second Hospital of Jilin University, Jilin University, Changchun, Jilin 130041, China

**Keywords:** Aortoesophageal fistula (AEF), Thoracic aortic aneurysm, Stent graft

## Abstract

Aortoesophageal fistula is a rare disease with a high mortality rate. The disease is with high mortality due to aneurysm rupture, and thus successfully managed cases are rarely reported. Here, we report a case of aortoesophageal fistula caused by a huge descending aneurysm and another smaller aneurysm found in the aortic arch. Such case was relatively rare in the cardiovascular field. Due to the limited experience, it was difficult to determine the proper therapeutic strategy. For this case, for the dual aneurysm, we surgically inserted an aortic endovascular stent-graft to exclusive the aneurysm and simultaneously repair the other aortic arch aneurysm. The patient had an uneventful recovery and was discharged after 1 month antibiotics therapy for the palliative treatment of the esophageal fistula. She survived for 8 months at home before dying of massive hematemesis. Here, we present the operative method and our therapeutic experience for this extremely rare case.

## Background

Aortoesophageal fistula (AEF) with thoracic aneurysms is very rare with poor prognosis, since most patients with AEF die of severe bleeding following aneurysm rupture. AEF is commonly caused by aortic or esophageal lesions. Approximately 80% of AEFs are caused by thoracic aortic aneurysms, trauma, and malignant esophageal tumors [1]. Most patients with thoracic aortic aneurysms are asymptomatic. Endoscopy is the preferred diagnostic method for identifying esophageal lesions. Endoscopy combined with enhanced computed tomography (CT) scanning was recommended to confirm the diagnosis of AEF and to determine the association between lesions [2].

The optimal management of AEF remains controversial, and much of the literature in this area has focused on the role of endovascular stent-graft implantation techniques [3-5]. However, the goal of all these interventions is to control massive hemorrhage and prevent aneurysm rupture, and then cure the esophageal fistula. The patient in this report had AEF combined with aneurysms in the descending aorta and aortic arch, which is an extremely rare case. The patient promptly underwent surgery that allowed direct access to the descending aorta for stent implantation to isolate the aneurysm and simultaneously repair the aortic arch aneurysm with an artificial graft patch. The patient was discharged after antibiotic therapy without any infection markers.

## Case presentation

**Figure 1:**
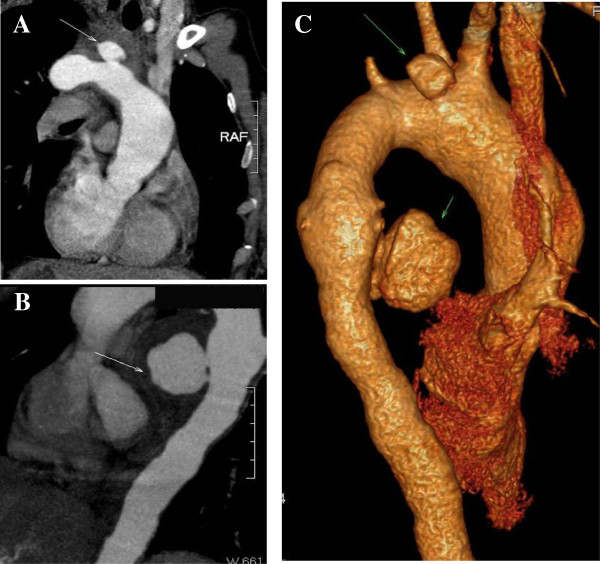
**Computed tomography angiography images of preoperation.** Its showing the aortic arch aneurysm **(A)** and descending aorta aneurysm **(B)**. A 3-dimensional aortic image **(C)** was constructed.

**Figure 2:**
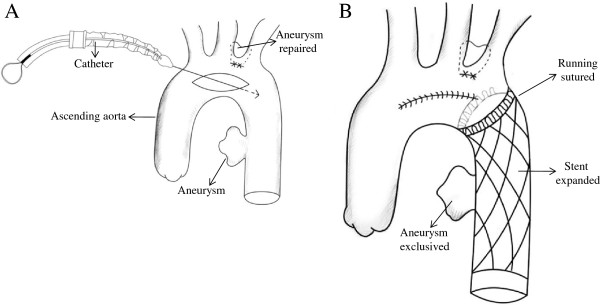
**Schematic diagram of the operation. (A)** Catheter inserting. **(B)** Stent graft implanted and incision closed.

**Figure 3:**
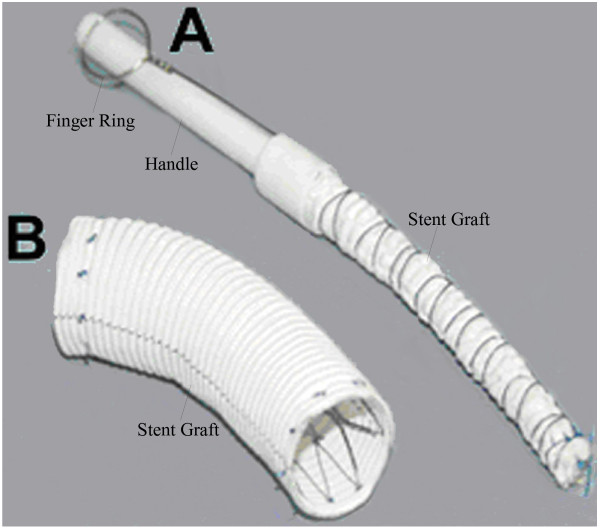
**Stented graft. (A) **Catheter structure and containing the stent graft in a bound and compressed state. **(B)** Stent graft expands completely (6).

A 34-year-old Chinese woman was treated in Second Hospital of Jilin University and survived for 8 months. She was admitted to the Department of Gastroenterology, the Second Hospital of Jilin University for a 15-day history of dysphagia after food intake and a 3-month history of intermittent chest pain. A bulge (2.0 × 2.0 cm^2^) with a smooth mucosal surface in the esophageal wall was observed by esophagoscopy at an external pressure of approximately 25 cm H_2_O. Aortic CT angiography (CTA) revealed two thoracic aortic aneurysms at the level of aortic arch (diameter, 2.7 cm), descending aorta, diffused descending aortic wall thickness and slightly dilation (diameter, 5.8 cm) of ascending aorta (Figure 1). After diagnostic confirmation was obtained, the patient was immediately transferred to the Department of Cardiovascular Surgery. Routine laboratory test results indicated that the patient had mild anemia and a slightly elevated white blood cell count. The next morning, the patient suffered from severe hematemesis with fresh blood. She immediately underwent emergency surgery for endovascular stent-graft placement. The surgical procedure consisted of a median sternotomy with cardiopulmonary bypass using right femoral artery and vena cava cannulation. Once the body temperature reduced to 25°C, circulation was arrested. A longitudinal incision was made on the lateral wall of the aortic arch. The selective cerebral perfusion was performed by innominate artery and left common carotid artery cannulation. The inner opening of the aortic arch aneurysm (diameter, 1 cm) was found between the innominate artery and the left common carotid artery, which was then repaired with a patch cut from prosthesis blood vessel (Figure 2A). A catheter sheath containing a stent graft (Microport Medical [Shanghai] Co, Ltd, China) of 26-mm diameter and 100 mm length stent-graft (Figure 3) was implanted into the descending aorta, and its proximal was far from the left subclavian artery (Figure 2B). The choice of appropriate sizing of the stent graft in patients was described in detail by Dr. Sun’s group [6]. The stent was running sutured with 5–0 Prolene to the descending aortic wall, and the aortic incision was closed.

**Figure 4:**
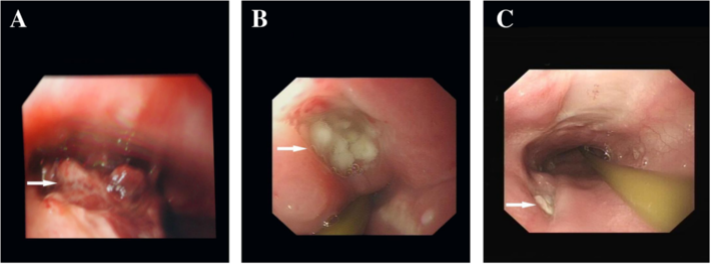
**Esophagogastroscopy images of postoperation.** Esophagogastroscopy showing an obvious ulcer and blood clot 1 day after surgery **(A)**. The esophageal ulcer was significantly reduced by postoperative day 25 days **(B)** and completely healed before discharge **(C)**.

The patient had a satisfactory postoperative recovery including circulatory, pulmonary, and central nervous system functions. On postoperative day 2, gastroendoscopy revealed an ulcer (diameter, 3 cm) was located approximately 25 cm from the incisors (Figure 4A). Esophageal stent was not suitable for the relatively large size of the ulcer. Since there were no obvious signs of mediastinal infection and pneumomediastinum, a conservative treatment protocol was selected for this patient. A feeding tube was inserted into the duodenum for nutritional administration.

**Figure 5:**
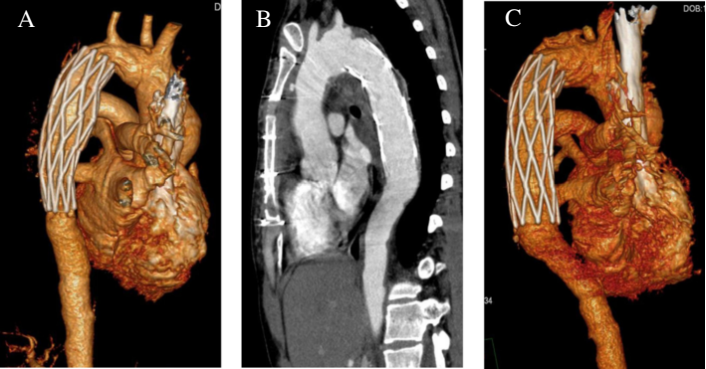
**Computed tomography angiography images of postoperation.** Its confirmed successful tamponade of the fistula before discharge **(A)** and after surgery for 6 months **(B and C)**.

Twenty-five days after surgery, gastroendoscopy revealed a significant reduction in the size of the ulcer (Figure 4B). An aneurysmal wall culture and three times blood culture was negative for any microorganisms. Meropenem which is a broad-specturem antibiotic was administered for 33 days until the no fever and leukocytosis was normal. CTA (Figure 5A) on postoperative day 36 showed that the position of the aortic stent was ideal and the aortic aneurysm was well closed without any abnormalities. Gastroendoscopy on postoperative day 45 (Figure 4C) showed that the ulcer was completely healed. The patient began oral soft food intake and she did not have any discomfort. The patient was discharged 1 week after initiation of normal diet intake.

Six months after surgery, aortic CTA showed that the position of aortic stent was also ideal without any abnormalities in the aorta (Figure 5B, C). Gastroendoscopy revealed that the esophageal damage was completely repaired.

Eight months after surgery, the patient was re-admitted urgently because of hemorrhagic shock caused by massive hematemesis. The patient was treated with fluid infusion, blood transfusion, and promptly emergency treatments. The CTA scan could not be done since severe shock of the patient, so aneurysm and esophageal lesion was not clear, and the reoperation would not be performed with the serious condition and unclear lesion change. All of these treatments failed and the patient died several hours later. Medical records indicate that the patient had a 1-month history of continuous low-grade fever for >1 month without any treatment. The cause of death was considered the aortic aneurysm rupture into the esophagus resulting from a vascular stent infection that led to hemorrhagic shock.

## Conclusion

AEF is a severe life-threatening condition with a high mortality rate [7]. After confirmation of diagnosis, patients should be treated immediately; otherwise, they may die of a hemorrhage or uncontrollable infection. In the past, AEF was managed with surgical thoracic esophagectomy and esophagogastrostomy to correct the fistula and esophagus. In addition to traditional open surgery for AEF treatment, endovascular stent-graft therapy is an effective method for preventing aortic rupture [8]. However, stent-graft therapy is associated with a high risk of spinal cord ischemia and other postoperative complications such as blood leaks, stenosis, and thrombosis within the stent or its migration [9].

In this case, the patient had aneurysms in both the descending aorta and the aortic arch. Thoracic endovascular aortic repair did not result in the closure of the aortic arch aneurysm. Therefore, we selected median sternotomy and stent placement to exclusive the descending aortic aneurysm and simultaneously repair another aortic arch aneurysm. We planned to place an esophageal stent to close the esophageal tear after repairing the aneurysm. However, the esophageal damage was too severe for stent treatment. No obvious signs of mediastinal infection were observed after surgery. Therefore, we chose to administer food through the duodenal tube to cure the esophageal ulcer. CTA and gastroendoscopy showed time-dependent recovery of the esophageal fistula without any infection and other complications. At 6 months of follow-up, CTA showed that the aortic stent position was fixed without dislodgement. However, the infection of the vascular stent caused reformation of the aortic aneurysm and rupture into the esophagus, leading to hemorrhagic shock and death 8 months after surgery.

Treatment of esophageal fistula is critical. Small esophageal fistulas without obvious infection can be treated by direct suturing [10]. However, cautious application is essential because of its association with a high risk of recurrent infection and mortality [3]. Infection, especially mediastinal infection, would cause problems for both surgery and interventional therapy. For patients with obvious mediastinal emphysema, simultaneous surgical debridement should be performed along with vascular replacement to reduce the risk of infection. After surgery, long-term treatment with appropriate antibiotics therapy is also necessary.

Secondary AEF is a fatal complication of thoracic endovascular aortic repair, although the incidence is only 1.6–1.9%. Emergent endovascular repair could increase the possibility of secondary AEF irrespective of the type of stent used [11]. Good short- and mid-term results were obtained for endovascular stent-graft implantation in many AEF management cases. Therefore, in patients with AEF, stent-graft implantation may be indicated only as an emergency management technique for controlling hemorrhage and avoiding AEF aggravation. Once the etiological factor of AEF is confirmed and the infection is controlled, a secondary surgery for esophageal reconstruction might be necessary for long-term survival.

In conclusion, open chest vascular stent implantation is an effective short-term treatment for AEF. This procedure could help surgeons to effectively exclusive the aneurysm and simultaneously repair other aortic lesions. In patients without mediastinal infections, conservative therapy with enteral nutrition through a duodenal tube is a simple and feasible modality for esophageal fistula. A second-stage operation for esophageal reconstruction may be performed once the patient can tolerate surgery. In addition, such patients need long-term antibiotic therapy even lift-long, and be strictly monitored blood tests and computed tomographic scans after discharge.

## Consent

Written informed consent was obtained from the patient for publication of this case report and any accompanying images. A copy of the written consent is available for review by the Editor-in-Chief of this journal.

## Abbreviations

AEF: Aortoesophageal fistula; CT: Computer tomography; CTA: Computerized tomographic angiography.

## Competing interests

The authors declare that they have no competing interests.

## Authors’ contributions

XCL and KXL wrote the manuscript. KXL, XRX and TCW performed the surgery. WY, ZCZ, DL and SDZ provided the information of the patient and performed literature search. All authors read and approved the final manuscript.
